# rMAP: the Rapid Microbial Analysis Pipeline for ESKAPE bacterial group whole-genome sequence data

**DOI:** 10.1099/mgen.0.000583

**Published:** 2021-06-10

**Authors:** Ivan Sserwadda, Gerald Mboowa

**Affiliations:** ^1^​ Department of Immunology and Molecular Biology, College of Health Sciences, School of Biomedical Sciences, Makerere University, Kampala, Uganda; ^2^​ Department of Biochemistry and Bioinformatics, School of Pure and Applied Sciences, Pwani University, Kilifi, Kenya; ^3^​ The African Center of Excellence in Bioinformatics and Data-Intensive Sciences, the Infectious Diseases Institute, College of Health Sciences, Makerere University, Kampala, Uganda

**Keywords:** command line, ESKAPE, pipeline, rapid microbial analysis, rMAP, whole-genome sequencing

## Abstract

The recent re-emergence of multidrug-resistant pathogens has exacerbated their threat to worldwide public health. The evolution of the genomics era has led to the generation of huge volumes of sequencing data at an unprecedented rate due to the ever-reducing costs of whole-genome sequencing (WGS). We have developed the Rapid Microbial Analysis Pipeline (rMAP), a user-friendly pipeline capable of profiling the resistomes of ESKAPE pathogens (*Enterococcus faecium*, *Staphylococcus aureus*, *Klebsiella pneumoniae*, *Acinetobacter baumannii*, *Pseudomonas aeruginosa* and *Enterobacter* species) using WGS data generated from Illumina’s sequencing platforms. rMAP is designed for individuals with little bioinformatics expertise, and automates the steps required for WGS analysis directly from the raw genomic sequence data, including adapter and low-quality sequence read trimming, *de novo* genome assembly, genome annotation, single-nucleotide polymorphism (SNP) variant calling, phylogenetic inference by maximum likelihood, antimicrobial resistance (AMR) profiling, plasmid profiling, virulence factor determination, multi-locus sequence typing (MLST), pangenome analysis and insertion sequence characterization (IS). Once the analysis is finished, rMAP generates an interactive web-like html report. rMAP installation is very simple, it can be run using very simple commands. It represents a rapid and easy way to perform comprehensive bacterial WGS analysis using a personal laptop in low-income settings where high-performance computing infrastructure is limited.

## Data Summary

1. The source code for single-nucleotide polymorphism (SNP) sites is available from GitHub under GNU GPL v3; (https://github.com/GunzIvan28/rMAP)
2. The authors confirm that all supporting data, code and protocols have been provided within the article. All sequencing reads from the exemplary data sets are publicly stored in the SRA database; accession IDs are provided.

**Impact Statement**

The evolution of the genomics era has led to the generation of massive chunks of sequencing data and different bioinformatics tools have been developed to analyse these data. The ever-reducing costs of whole-genome sequencing (WGS) have led to diagnostic and research laboratories obtaining genome sequencing technologies. The considerable bioinformatics skills needed to analyse the large volume of genomic data from these platforms and the complex format in which results are presented offer two important impediments in the implementation of WGS. To the best of our knowledge, there is currently no published all-in-one bioinformatics tool that successfully provides: genome-assembly statistics; single-nucleotide polymorphism (SNP) variant calling; phylogenetic analysis; antimicrobial resistance, plasmid and virulence factor profiling; multi-locus sequence typing; pangenome analysis; and insertion sequence characterization (IS) for ESKAPE pathogens. Therefore, we introduce rMAP (https://github.com/GunzIvan28/rMAP), a rapid microbial analysis pipeline for comprehensive analysis of bacterial WGS data. This is an open-source, user-friendly, command-line and scalable pipeline for conducting WGS analysis of Illumina sequencing reads. It represents a rapid and easy way to perform comprehensive bacterial WGS analysis using personal laptops, especially in low-income settings where high-performance computing infrastructure is limited. rMAP generates a web-like html interactive report (https://gunzivan28.github.io/rMAP/) that can be shared and interpreted by microbiologists.

## Introduction

The recent re-emergence of multidrug-resistant pathogens through persistent misuse of antibiotics has exacerbated their threat to worldwide human public health and well-being. Such organisms, consisting of *Staphylococcus aureus*, *Pseudomonas aeruginosa* and *Klebsiella* species belonging to the ESKAPE pathogen group, have been flagged among the most notorious micro-organisms expressing tremendously high levels of antimicrobial resistance by the World Health Organization (WHO), and have been reported by many studies to contribute to the high frequency of nosocomial infections which have led to high morbidity and mortality rates all over the world [1–3].

In the same spirit, rapid advances in diagnostic science and personalized medicine have seen the emergence of high-throughput next-generation sequencing technologies to replace conventional microbiology laboratories, and this has greatly reduced diagnostic costs and turnaround times for results for infectious pathogens as a way of keeping pace with emerging multidrug-resistant varieties. Next-generation processes generally involve parallel sequencing, producing vast quantities of genomic data, and extensive modern computation infrastructure is required to make sense of the sequencing data in downstream analysis. Furthermore, another bottleneck in the deployment of high-throughput sequencing (HTS) technologies is the ability to analyse the increasing amount of data produced in a fit-for-purpose manner [4]. The field of microbial bioinformatics is thriving and quickly adapting to technological changes, which creates difficulties for clinical microbiologists with little or no bioinformatics background in following the complexity and increasingly obscure jargon of this field [4].

The routine application of whole-genome sequencing (WGS) requires cheap, user-friendly techniques that can be used on-site by personnel who have not specialized in big data management [5, 6]. The ability of bioinformaticists to analyse, compare, interpret and visualize the vast increase in bacterial genomes is valiantly trying to keep up with these developments [7]. Many biologists are drowning in too much data, and in desperate need of a tool capable of deciphering this complex information, and it is predicted that these trends will continue in the foreseeable future as the generation of genome data becomes cheaper and abundant [7].

Therefore, we introduce the Rapid Microbial Analysis Pipeline (rMAP), a one-stop toolbox that uses WGS illumina data to characterize the resistomes of bacteria of ESKAPE origin. This is an open-source, user-friendly, command-line, automated and scalable pipeline for conducting analysis of HTS data produced by Illumina platforms. rMAP takes raw sequencing data as input and performs bacterial bioinformatic analysis steps, including: adapter and low-quality sequence trimming, *de novo* genome assembly, genome annotation, SNP variant calling, phylogenetic inference by maximum likelihood, antimicrobial resistance profiling, plasmid profiling, virulence factor determination, multi-locus sequence typing (MLST), pangenome analysis and insertion sequence (IS) characterization.

## Methods

### Pipeline architecture

rMAP is a tool implemented in four programming languages, namely Shell script, Python, Perl and R. It was precompiled and supports the Linux 64-bit architecture and macOS version 10.14.6 (Mojave) and above. It was originally built using WSL Ubuntu 20.04.1 LTS (Focal Fossa) and Ubuntu 18.04.4 LTS (Bionic Beaver) and the binaries are compatible with noarch–Unix-style operating systems.

rMAP was built using a collection of published reputable tools such as FASTQC [8], MultiQC [9], Trimmomatic [10], Shovill, Megahit [11], Prokka [12], Freebayes, SnpEff [13], IQtree [14], BWA [15], Samtools [16], Roary [17] and ISMapper [18], just to mention a few. All of the tools and third-party dependences required by rMAP are resolved and containerized within a conda environment as a single package so as not to interfere with already existing programs. The programs in the conda environment are built on top of Python version 3.7.8 [19] and are compatible with R statistical package version 4.0.2 [20]. A full list of the packages used by rMAP is provided in Table 1.

**Table 1.** Comprehensive list of third-party tools and algorithms used in rMAP

Software	Version	Summary
Abricate	1.0.1	Detection of antimicrobial resistance genes, plasmids and virulence factors
AMRfinder	3.8.4	Detection of antimicrobial resistance genes from assembled contigs
Any2fasta	0.4.2	Converts any genomic data format to fasta format
Assembly-stats	1.0.1	Summarizes quality assembly metrics from contigs
Biopython.convert	1.0.3	Conversion and manipulation of different genomic data formats
BMGE	1.12	Block mapping and gathering with entropy for removal of ambiguously aligned reads from multiple sequence alignments
BWA	0.7.17	Burrow–Wheeler algorithm for fast alignment of short sequence reads
Cairosvg	2.4.2	Converts SVG to PDF and PNG formats
Fastqc	0.11.9	Quality control and visualization of HTS data
Fasttree	2.1.10	Ultra-fast inference of phylogeny using the maximum-likelihood method
Freebayes	1.3.2	Bayesian-based haplotype prediction of nucleotide variants
ISMapper	2.0.1	Detection of insertion sequences within genomes
IQtree	2.0.3	Inference of phylogeny using the maximum-likelihood method
Kleborate	1.0.0	Screening for AMR genes and MLSTs from genome assemblies
Lxml	4.5.2	Parsing of XML and HTML using Python
Mafft	7.471	Algorithm for performing multiple sequence alignments
Multiqc	1.9	Aggregates numerous HTML quality reports into a single file
Megahit	1.2.9	Ultra-fast genome assembly algorithm
Mlst	2.19.0	Characterization and detection of clones within a population of pathogenic isolates
Nextflow	20.07.1	Portable next-generation workflow language that enables reproducibility and development of pipelines
Parallel	20200722	Executes jobs in parallel
Prinseq	0.20.4	Trims, filters and reformats genomic sequence data
Prodigal	2.6.3	Prediction of protein-coding genes in prokaryotic genomes
Prokka	1.14.6	Fast and efficient annotation of prokaryotic assembled genomes
Quast	5.0.2	Quality assembly assessment tool
Roary	3.13.0	Large-scale pangenome analysis
R-base	4.0.2	Statistical data computing and graphical software
Samclip	0.4.0	Filters SAM file for soft and hard clipped alignments
Samtools	1.9	Tools for manipulation of next-generation sequence data
Shovill	1.0.9	Illumina short-read assembler for bacterial genomes
Snippy	4.3.6	Rapid haploid bacterial variant caller
Snpeff	4.5covid19	Functional effect and variant predictor suite
SRA-tools	2.10.8	Toolbox for acquisition and manipulation of sequences from the NCBI
Trimmomatic	0.39	Illumina short-read adapter trimming algorithm
Unicycler	0.4.8	A hybrid assembly pipeline for Illumina and long-read sequence data
Vt	2015.11.10	A tool for normalizing variants in genomic sequence data

### Overview of rMAP workflow

rMAP can be used with an unlimited number of samples of different species and origins. However, it was built to target pathogens of public health concern exhibiting high levels of antimicrobial resistance (AMR) and nosocomial infections. It can be applied to isolates of human and animal origin to give insights into the transmission dynamics of AMR genes at the human–animal interface.

### Benchmarking datasets

The pipeline was tested on numerous bacterial pathogens from the ESKAPE group isolated from different origins (clinical, faecal, animal and sewage), sequenced on Illumina platforms and obtained from the publicly available repositories the Sequence Read Archive (SRA) and the European Nucleotide Archive (ENA) under the following accessions: *Enterococcus* species (SRR8948878, SRR8948879, SRR8948880, SRR8948881, SRR8948882, SRR8948883, SRR8948884, SRR8948885, SRR8948886, SRR8948887, SRR8948888, SRR8948889, SRR8948890, SRR8948891), *Acinetobacter baumannii* (ERR1989084, ERR1989100, ERR1989115, ERR3197698, SRR3666962, SRR5739056, SRR6037664, SRR8289559, SRR8291681), *Klebsiella* species (SRR8753739, SRR8753737, SRR8291573, SRR11816972, SRR9703249, SRR9029107, SRR9029108, SRR8610335, SRR8610353, SRR8610357, SRR8610351, SRR8610354, SRR9964283, SRR9044171, SRR5687278, SRR5514226, SRR5514224, SRR5514223) and *Staphylococcus aureus* (ERR1794900, ERR1794901, ERR1794902, ERR1794903, ERR1794904, ERR1794905, ERR1794906, ERR1794907, ERR1794908, ERR1794909, ERR1794910, ERR1794911, ERR1794912, ERR1794913, ERR1794914). The GenBank references used include *A. baumannii* strain 36–1512, accession CP059386.1, GI 1880620189; *Enterococcus faecalis* strain KB1, accession CP015410.2, GI 1173533644; and *S. aureus* subsp. aureus strain MRSA252, accession BX571856.1, GI 49240382.

### Core pipeline features

rMAP requires three mandatory parameters; the input directory that contains sequence reads in either fastq or fastq.gz formats, an output user-defined directory and a reference genome in either GenBank or fasta format. A full GenBank reference genome file is recommended for the --reference option to obtain an annotated VCF files. The raw fastq files are directly submitted to rMAP, with no prior bioinformatics treatment, as follows:

rMAP -t 8 --reference --input dir_name--output dir_name --quality --assembly megahit --amr --varcall --phylogeny --pangenome --gen-ele

**Fig. 1.**
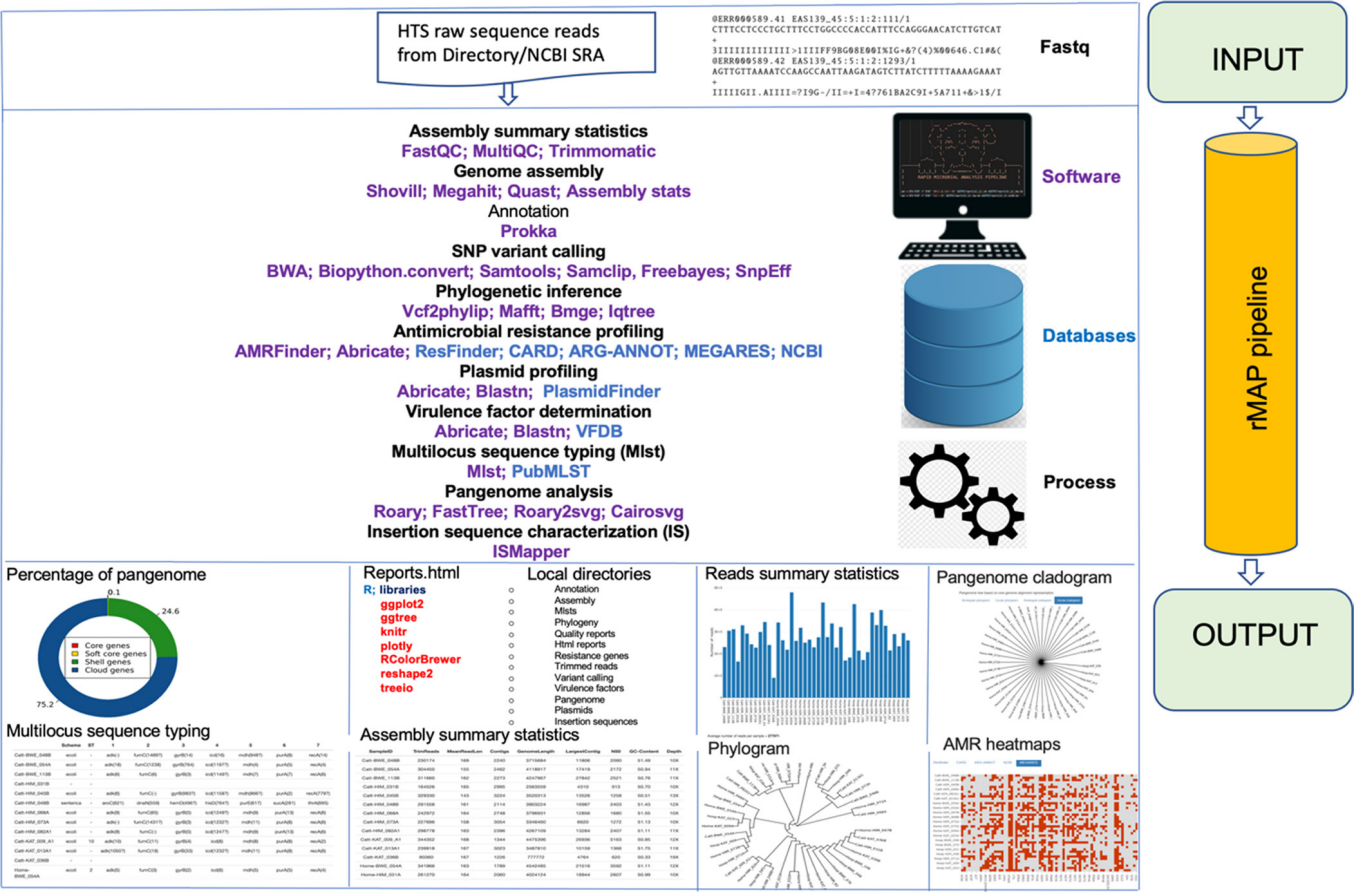
Schematic graphical representation of rMAP pipeline workflow and associated tools.

The pipeline’s features can be summarized in the order of: SRA sequence download, quality control, adapter trimming, *de novo* assembly, resistome profiling, variant calling, phylogenetic inference, pangenome analysis, insertion sequence mapping and report generation, as shown in Fig. 1.

### Sequence read archive download

rMAP is able to retrieve sequences from the NCBI’s SRA using fastq-dump [21]. A user simply creates a list containing the sample accession numbers to be downloaded saved at the home directory. The downloaded sequences are saved in a default directory called SRA-READS created by rMAP.

### Quality assessment and filtering

The pipeline autodetects any non-zipped fastq reads and parses them to the fastq.gz format for optimization purposes during downstream analysis. Fastqc [8] generates sequence quality reports and statistics from each individual sample, which are then aggregated into a single graphically interactive html report using MultiQC [9].

### Adapter and low sequence read trimming

Trimmomatic [10] is used to trim off adapters using a set of pre-defined Illumina library preparation adapters saved in fasta format and low sequence regions from the raw input sequence reads. The pipeline’s default parameters for quality and minimum sequence length are set at a phred quality score of 27 and 80 base pairs, respectively, to accommodate sequencing data that may not be of the very high recommended quality (i.e. 33).

### *De novo* assembly and annotation

Two assemblers are selected for this purpose for a user to choose from – Shovill [22] and Megahit [11] – each demonstrating an advantage over the other. Both algorithms take the trimmed reads as their input and perform k-mer-based assembly to produce contigs. Megahit exhibited very fast computational speeds, almost half those of its counterpart, but with slightly lower quality assembly metrics. Assembly with Shovill involves guided mapping of the contigs to a reference and numerous rounds of genome polishing using pilon to remove gaps,and takes more time but produces good quality assembly metrics (N50, L50, genome length). Prodigal [23] is used to predict open reading frames from the assembled contigs, which are then functionally annotated using Prokka [12].

### Variant calling

The trimmed reads are aligned against a an indexed reference in the fasta format using the Burrows–Wheeler aligner [15] to produce SAM files. Soft and hard clipped alignments are removed from the sequence alignment map (SAM) files using Samclip (https://github.com/tseemann/samclip). Samtools [16] then sorts, marks duplicates and indexes the resultant binary alignment map (BAM) files. Freebayes [24] calls variants using Bayesian models to produce variant call format (VCF) files containing single-nucleotide polymorphism (SNP) information, which is filtered using bcftools (https://github.com/samtools/bcftools) and normalized of biallelic regions using Vt [25]. The filtered VCF files are annotated using snpEff [13]. Raw, tab-separated, annotated and filtered VCF files are available for the users to manipulate.

### Resistome profiling

The conceptualization of rMAP was aimed at exhaustively exploiting the resistome of pathogenic bacteria. AMRfinder plus [26] predicts resistance genes using its database. Mass screening for antimicrobial resistance genes is performed using the CARD [27], ARG-ANNOT [28], NCBI, ResFinder and MEGARES [29] databases. Plasmids and virulence factors are typed from the assembled genomes using PlasmidFinder [30] and the Virulence Factor Database (VFDB) [31], respectively, using Abricate (https://github.com/tseemann/abricate). Multi-locus sequence typing is performed using Mlst (https://github.com/tseemann/mlst).

### Phylogenetic inference

Because of the computationally demanding requirements of algorithms in terms of RAM and core threads during phylogenetic analysis, rMAP incorporates the use of SNP-based analysis, which has been proven to be faster than using sequencing data to infer phylogeny. A single VCF file containing all the samples and their SNPs is generated towards the end the variant calling stage, which is transposed by vcf2phylip [32] into a multi-alignment fasta file. Multi-sequence alignment is performed using Mafft [33], with the removal of ambiguously aligned reads and the selection of informative regions to infer phylogeny using BMGE [34]. IQtree [14] tests various substitution models and constructs trees from the alignments using the maximum-likelihood method with 1000 bootstraps. The resulting trees are visualized in rectangular (phylogram), circular (phylogram) and circular (cladogram) forms.

### Pangenome analysis

Roary [17] is employed by rMAP to perform core and accessory pangenome analysis across the input samples using general feature format (.gff) files generated from the annotation step. Fasttree is used to convert the core genome alignment to the newick format. The scalable vector graphic (SVG) file obtained from the pangenome analysis is converted to a portable network graphic (PNG) file format by cairosvg (https://cairosvg.org/). The resulting trees are visualized in rectangular (phylogram), circular (phylogram) and circular (cladogram) forms.

### Insertion sequence (IS) analysis

rMAP interrogates for the presence of mobile genetic elements, in particular insertion sequences, using ISMapper [18], which basically spans the lengths of the entire genome of a sequence searching for homology against a set of well-known insertion sequence families commonly found in ESKAPE isolates [35] and the ISfinder database (https://www-is.biotoul.fr/index.php), as shown in Table 2.

**Table 2.** ESKAPE group insertion sequence families (both Gram-positive and Gram-negative) used by rMAP

Sequence name	Determinant genes	Conferred resistance
IS903	*aphA1*	Kanamycin
ISApl1	*mcr-1*	Colistin
ISEc69	*mcr-2*	Colistin
ISAba14	*aphA6*	Kanamycin
ISAba1	*blaOXA-23*	Carbapenems, beta-lactams
IS16	*VanB1*	Vancomycin
IS256	*cfr*	Phenicols, lincosamides, oxazolidinones, pleuromutilins, streptogramin A
IS257-2	*aadD*, *ble*, *fosB5*, *fusB*, *tetL*, *tetK*, *aacA-aphD*, *vatA*, *dfrK*	Kanamycin, bleomycin, fosfomycin, fusidic acid, tetracycline, gentamicin, streptogramin A, trimethoprim
IS1182	*aadE*, *aphaA-3*, *sat4*	Streptomycin, kanamycin, neomycin, streptothricin
IS1216	*cfr*, *str*	Phenicols, lincosamides, oxazolidinones, pleuromutilins, streptomycin, streptogramin A
IS1272	*mecR*	Methicillin
IS1182	*aphA-3, aadE*	Aminoglycoside
ISEnfa4	*cfr*	Phenicols, lincosamides, oxazolidinones, pleuromutilins, streptogramin A
ISEcp1	*CTX-M*	Cefotaxime, ceftriaxone, aztreonam
ISSau1	*SCCmec*	Methicillin
ISKpn23	*blaBKC-1*	Carbapenems, cephalosporins, monobactams

## Results

### Reporting and visualization of the reports

**Fig. 2.**
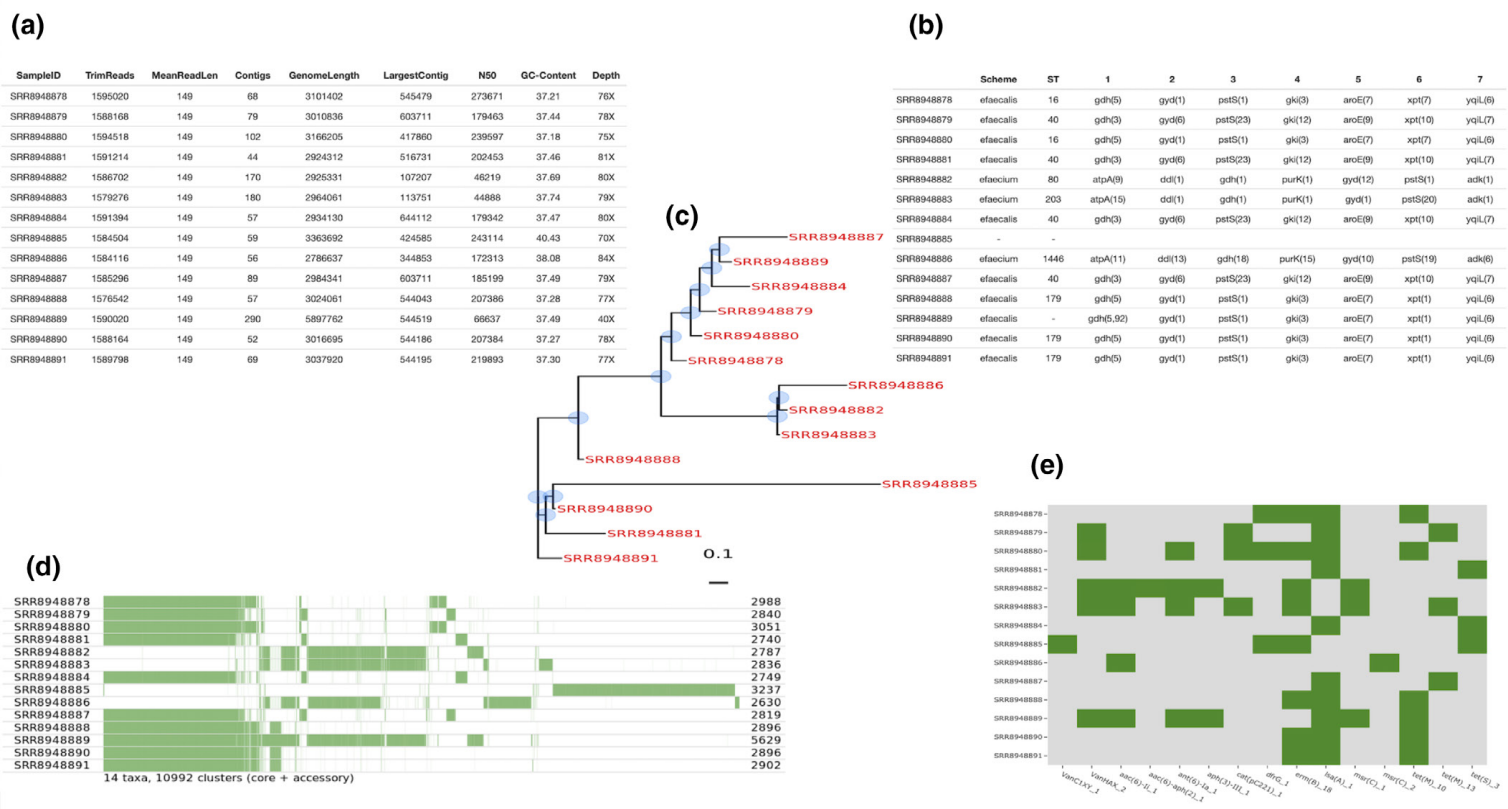
Selected interactive *Enterococcus* species HTML reports. (**a**) Genome assembly summary statistics for the different *Enterococcus* species isolates. These include common genome analysis key metrics for checking assembly quality. (**b**) Table of multi-locus sequence typing (MLST) distribution. (**c**) SNP-based approximately maximum-likelihood phylogenetic tree. Three different formats are available, i.e circular (phylogram), circular (cladogram) and rectangular (phylogram). An approximately maximum-likelihood phylogenetic tree is computed based on SNPs detected via read mapping against a reference genome and stored in a standard Newick file format. (**d**) Pangenome analysis including a schematic representation of gene presence (colour) or absence (blank) between samples. (**e**) Antibiotic resistance profile. Presence/absence of antibiotic resistance genes (coverage and identity >90 %) for each sample. An antibiotic resistance profile is computed based on Resfinder, CARD, ARG-ANNOT, NCBI and MEGARES annotations for each isolate and transformed into an overview that allows a rapid resistome comparison of all analysed isolates.

rMAP stores and formats reports from each stage of the pipeline under one directory called ‘reports’ and uses R-base [20] with a set of R packages, including ggtree [36], RcolorBrewer, ggplot2 [37], knitr [38], rmarkdown [39], plotly [40], reshape2 [41] and treeio [42], to generate a web-like html interactive report with explanations at every stage of analysis that can easily be shared and interpreted by inexperienced bioinformatics individuals. An example of such a report can be accessed via https://gunzivan28.github.io/rMAP/. The reporting format for rMAP was mainly adapted from the Tormes [6] pipeline. The results from a successful run can be found under the user-defined output directory and consist of files from assembly, annotation, insertion sequences, mlsts, pangenomes, phylogeny, plasmids, quality reports, quast assembly stats, reports, resistance genes, trimmed reads, variant calling and virulence factors for further analysis. rMAP retains all of the intermediate files generated after a successful run to be interrogated further by experienced bioinformatics users. The contents extracted from the intermediate files and summarized in the html report with a short description are summarized in Table 3. Examples of visuals generated by the pipeline are illustrated in Fig. 2.

**Table 3.** Summary of some stages of intermediate files generated from rMAP

Analysis	Metrics	Description
Assembly	Genome length, average genome length, N50, GC content and sequencing depth	Genome length – an estimate of the draft genome assembly length Average genome length – average read length of genomes N50 – length of smallest contig covering 50 % of genome GC content – guanine–cytosine content of draft genome Depth – no. of times each nucleotide position in the draft genome has a read that aligns to that position
Phylogeny		SNPs are used to infer phylogenetic relationships between samples
Variant calling	SNPs	SNP – a single-nucleotide base change from the reference genome that occurs anywhere within the genome
Antimicrobial resistance profiling	Contig, gene, identity, product	Contig – continuous consensus nucleotide sequences without gaps Gene – antibiotic resistance gene identified within the assembly Identity – percentage representing exact nucleotide matches Product – artefact produced from antibiotic resistance gene
Pangenome analysis	Core genes, soft core genes, shell genes, cloud genes	The genes are compared against each other across samples to predict genome plasticity and to detect how much of the accessory genome has been taken up by organisms over the course of time

### Computational infrastructure and benchmarking

The original philosophy of creating rMAP was to create a tool that can be easily installed and run on a descent personal computer. The pipeline was successfully compiled on two personal computers with the following specifications: Dell Inspiron 5570 8th Gen Intel Core i7-8550U CPU @1.80 GHz (8 CPUs), ~2.0 GHz with 12 GB of RAM and 1 TB of hard disk space running Windows subsystem Linux (WSL) Ubuntu 20.04.1 LTS (Focal Fossa) and Ubuntu 18.04.4 LTS (Bionic Beaver) and a MacBook pro Intel Core i7 CPU @3.0 GHz, 16 GB of RAM and 2 TB of SSD space running on macOS Mojave. When provided with the same samples, the MacBook performed better because of its hardware compared to Ubuntu. Depending on the number of samples provided in the input, rMAP generates intermediate files ranging between 10 and 30 GB. The wall clock runtimes and benchmarking statistics for each bacterial species on different platforms are summarized in Table 4.

**Table 4.** rMAP’s wall clock runtimes for different bacterial species across different operating system platforms

Genomes	Genome size	Ubuntu	macOS Mojave
15 *Staphylococcus aureus*	~2.9 Mbp	22 h	18 h
9 *Acinetobacter baumannii*	~3.9 Mbp	22 h	19 h
14 *Enterococcus* spp.	~2.9 Mbp	21 h	17 h

## Discussion

Although other pipelines developed under the same philosophy and functionality as rMAP, such as Tormes [6], ASA3P [43] and the recently published Bactopia [44], exist, we noticed that each of these had a shortcoming that we aimed to address. In terms of usability, Tormes [6] was the most friendly pipeline, with one major drawback, where it could never be launched without a tab-separated metadata file complying with a set criteria. It was also more oriented to bacterial species-specific analyses, namely *Escherichia coli* and *Salmonella* species. ASA3P [43] and Bactopia [44] required a bioinformatics-competent user for operation, since they are written in complex languages, namely, Groovy and Nextflow, respectively. Other similar pipelines, such as Nullarbor (https://github.com/tseemann/nullarbor), were extremely difficult to compile and use compared to their counterparts, requiring a metadata file conforming with set criteria. In cases where metadata files are required, the different software flagged errors or halted task executions as the correct conforming metadata files were essential for the downstream analyses.

rMAP, on the other hand, comes with features aimed at overcoming the limitations of its counterparts. It requires no prior preprocessing of the sequences or metadata files. The user only provides three essential requirements, namely, an input directory, an output directory and a reference genome to run the pipeline. The pipeline is written in basic programming languages that do not require advanced expertise or troubleshooting to be launched. rMAP is highly portable and capable of operating on decent personal computers running on either Ubuntu or macOS. Installation is quite easy and straightforward from the GitHub repository (https://github.com/GunzIvan28/rMAP), with the binaries and dependences built within conda environment packages. Most of all, rMAP shows a high sensitivity towards analysis and is not limited to the ESKAPE group pathogens, but also applies to other *Enterobacteria*, such as *E. coli* and *Salmonella* species.

As a significant limitation, rMAP is coded exclusively in Bash and is not implemented within a modern workflow language manager, such as Snakemake or Nextflow. The ultimate consequence of this is that a user will have to either restart the whole run or manually check which steps have completed successfully and resume the run by only selecting options that were not performed while excluding the computed steps from the main command script. Implementation of the pipeline within a modern workflow language will feature in the next release of the software.

## Conclusion

rMAP is a robust, scalable, user-friendly, automated bioinformatics analysis workflow for Illumina WGS reads that has demonstrated efficiency in the analysis of public health-significant pathogens. Therefore, we recommend it as a tool for continuous monitoring and surveillance that is suitable for assessing antimicrobial resistance gene trends, especially in low-income countries with limited computational bioinformatics infrastructure.

### Availability and future directions

The source code is available on GitHub under a GPL3 licence at https://github.com/GunzIvan28/rMAP. Questions and issues can be sent to ivangunz23@gmail.com and bug reports can be filed as GitHub issues. Although rMAP itself is published and distributed under a GPL3 licence, some of its dependences bundled within the rMAP volume are published under different licence models.

## References

[1] Sserwadda I, Lukenge M, Mwambi B, Mboowa G, Walusimbi A (2018). Microbial contaminants isolated from items and work surfaces in the post- operative ward at Kawolo General Hospital, Uganda. BMC Infect Dis.

[2] Mulani MS, Kamble EE, Kumkar SN, Tawre MS, Pardesi KR (2019). Emerging strategies to combat ESKAPE pathogens in the era of antimicrobial resistance: a review. Front Microbiol.

[3] Ma Y-X, Wang C-Y, Li Y-Y, Li J, Wan Q-Q, Chen J-H (2020). Considerations and caveats in combating ESKAPE pathogens against nosocomial infections. Adv Sci.

[4] Carriço JA, Rossi M, Moran-Gilad J, Van Domselaar G, Ramirez M (2018). A primer on microbial bioinformatics for nonbioinformaticians. Clin Microbiol Infect.

[5] Hyeon J-Y, Li S, Mann DA, Zhang S, Li Z (2018). Quasimetagenomics-based and real-time-sequencing-aided detection and subtyping of *Salmonella enterica* from food samples. Appl Environ Microbiol.

[6] Quijada NM, Rodríguez-Lázaro D, Eiros JM, Hernández M (2019). TORMES: an automated pipeline for whole bacterial genome analysis. Bioinformatics.

[7] Land M, Hauser L, Jun S-R, Nookaew I, Leuze MR (2015). Insights from 20 years of bacterial genome sequencing. Funct Integr Genomics.

[8] Andrews S (2010). FastQC: a Quality Control Tool for High Throughput Sequence Data.

[9] Ewels P, Magnusson M, Lundin S, Käller M (2016). MultiQC: summarize analysis results for multiple tools and samples in a single report. Bioinformatics.

[10] Bolger AM, Lohse M, Usadel B (2014). Trimmomatic: a flexible trimmer for illumina sequence data. Bioinformatics.

[11] Li D, Liu C-M, Luo R, Sadakane K, Lam T-W (2015). MEGAHIT: an ultra-fast single-node solution for large and complex metagenomics assembly via succinct de Bruijn graph. Bioinformatics.

[12] Seemann T (2014). Prokka: rapid prokaryotic genome annotation. Bioinformatics.

[13] Cingolani P, Platts A, Wang LL, Coon M, Nguyen T (2012). A program for annotating and predicting the effects of single nucleotide polymorphisms, SnpEff. Fly.

[14] Nguyen L-T, Schmidt HA, von Haeseler A, Minh BQ (2015). IQ-TREE: a fast and effective stochastic algorithm for estimating maximum-likelihood phylogenies. Mol Biol Evol.

[15] Li H, Durbin R (2009). Fast and accurate short read alignment with Burrows-Wheeler transform. Bioinformatics.

[16] Li H, Handsaker B, Wysoker A, Fennell T, Ruan J (2009). The sequence Alignment/Map format and SAMtools. Bioinformatics.

[17] Page AJ, Cummins CA, Hunt M, Wong VK, Reuter S (2015). Roary: rapid large-scale prokaryote pan genome analysis. Bioinformatics.

[18] Hawkey J, Hamidian M, Wick RR, Edwards DJ, Billman-Jacobe H (2015). ISMapper: identifying transposase insertion sites in bacterial genomes from short read sequence data. BMC Genomics.

[19] Rossum G (1995). Python reference manual.

[20] Ihaka R, RJJoc G (1996). Statistics G. R: a language for data analysis and graphics..

[21] ncbi/sra-tools (2020). NCBI - National Center for Biotechnology Information/NLM/NIH.

[22] Seemann T (2020). Tseemann/shovill. https://github.com/tseemann/shovill.

[23] Hyatt D, Chen G-. L, LoCascio PF, Land ML, Larimer FW (2010). Prodigal: prokaryotic gene recognition and translation initiation site identification. BMC Bioinformatics.

[24] Garrison E, Marth G (2012). Haplotype-based variant detection from short-read sequencing. arXiv:12073907 [q-bio].

[25] Tan A, Abecasis GR, Kang H (2015). Unified representation of genetic variants. Bioinformatics.

[26] Feldgarden M, Brover V, Haft DH, Prasad AB, Slotta DJ (2019). Validating the AMRFinder tool and resistance gene database by using antimicrobial resistance genotype-phenotype correlations in a collection of isolates. Antimicrob Agents Chemother.

[27] McArthur AG, Waglechner N, Nizam F, Yan A, Azad MA (2013). The comprehensive antibiotic resistance database. Antimicrob Agents Chemother.

[28] Gupta SK, Padmanabhan BR, Diene SM, Lopez-Rojas R, Kempf M (2014). ARG-ANNOT, a new bioinformatic tool to discover antibiotic resistance genes in bacterial genomes. Antimicrob Agents Chemother.

[29] Doster E, Lakin SM, Dean CJ, Wolfe C, Young JG (2019). MEGARes 2.0: a database for classification of antimicrobial drug, biocide and metal resistance determinants in metagenomic sequence data. Nucleic Acids Res..

[30] Carattoli A, Zankari E, García-Fernández A, Larsen MV, Lund O (2014). In silico detection and typing of plasmids using PlasmidFinder and plasmid multilocus sequence typing. Antimicrob Agents Chemother.

[31] Liu B, Zheng D, Jin Q, Chen L, Yang JJ (2019). Nar. VFDB 2019: a comparative pathogenomic platform with an interactive web interface. Nucleic Acids Res.

[32] Ortiz E (2018). vcf2phylip V1. 5: convert a VCF matrix into several matrix formats for phylogenetic analysis. Zenodo.

[33] Katoh K, Standley DMJMb, evolution (2013). MAFFT multiple sequence alignment software version 7: improvements in performance and usability. Mol Biol Evol.

[34] Criscuolo A, Gribaldo S (2010). BMGE (block mapping and Gathering with entropy): a new software for selection of phylogenetic informative regions from multiple sequence alignments. BMC Evol Biol.

[35] Partridge SR, Kwong SM, Firth N, Jensen SO, SOJCmr J (2018). Mobile genetic elements associated with antimicrobial resistance. Clin Microbiol Rev.

[36] Yu G, Smith DK, Zhu H, Guan Y, Lam TT-Y (2017). ggtree: an R package for visualization and annotation of phylogenetic trees with their covariates and other associated data. Methods Ecol Evol.

[37] Wickham H (2016). ggplot2: Elegant Graphics for Data Analysis.

[38] Xie Y (2015). Dynamic documents with R and knitr.

[39] Grolemund G, Allaire JJ, Xie Y (2018). R Markdown: the definitive guide.

[40] Plotly https://plotly.com/r/.

[41] Wickham H (2007). Reshaping data with the reshape package. J Stat Softw.

[42] Yu G, Smith DK, Zhu H, Guan Y, Lam TTY (2017). ggtree: an R package for visualization and annotation of phylogenetic trees with their covariates and other associated data. Methods Ecol Evol.

[43] Schwengers O, Hoek A, Fritzenwanker M, Falgenhauer L, Hain T (2020). ASA3P: an automatic and scalable pipeline for the assembly, annotation and higher-level analysis of closely related bacterial isolates. PLoS Comput Biol.

[44] Petit RA, Read TD (2020:2020.02.28.969394). Bactopia: a flexible pipeline for complete analysis of bacterial genomes. bioRxiv.

